# Elucidation of Arctigenin Pharmacokinetics and Tissue Distribution after Intravenous, Oral, Hypodermic and Sublingual Administration in Rats and Beagle Dogs: Integration of *In Vitro* and *In Vivo* Findings

**DOI:** 10.3389/fphar.2017.00376

**Published:** 2017-06-14

**Authors:** Jie Li, Xin Li, Yu-Shan Ren, Yuan-Yuan Lv, Jun-Sheng Zhang, Xiao-Li Xu, Xian-Zhen Wang, Jing-Chun Yao, Gui-Min Zhang, Zhong Liu

**Affiliations:** ^1^Shandong New Time Pharmaceutical Co., LtdLinyi, China; ^2^Center for New Drug Safety Evaluation of Lunan Pharmaceutical, Lunan Pharmaceutical Group Co., LtdLinyi, China; ^3^State Key Laboratory of Generic Manufacture Technology of Chinese Traditional Medicine, Lunan Pharmaceutical Group Co., LtdLinyi, China

**Keywords:** arctigenin, pharmacokinetics, UPLC/MS/MS, bioavailability, microsomes

## Abstract

Although *arctigenin* (*AG*) has diverse bioactivities, such as anti-oxidant, anti-inflammatory, anti-cancer, immunoregulatory and neuroprotective activities, its pharmacokinetics have not been systematically evaluated. The purpose of this work was to identify the pharmacokinetic properties of AG via various experiments *in vivo* and *in vitro*. In this research, rats and beagle dogs were used to investigate the PK (pharmacokinetics, PK) profiles of AG with different drug-delivery manners, including intravenous (i.v), hypodermic injection (i.h), and sublingual (s.l) administration. The data shows that AG exhibited a strong absorption capacity in both rats and beagle dogs (absorption rate < 1 h), a high absorption degree (absolute bioavailability > 100%), and a strong elimination ability (*t*_1/2_ < 2 h). The tissue distributions of AG at different time points after i.h showed that the distribution of AG in rat tissues is rapid (2.5 h to reach the peak) and wide (detectable in almost all tissues and organs). The AG concentration in the intestine was the highest, followed by that in the heart, liver, pancreas, and kidney. *In vitro*, AG were incubated with human, monkey, beagle dog and rat liver microsomes. The concentrations of AG were detected by UPLC-MS/MS at different time points (from 0 min to 90 min). The percentages of AG remaining in four species’ liver microsomes were human (62 ± 6.36%) > beagle dog (25.9 ± 3.24%) > rat (15.7 ± 9%) > monkey (3.69 ± 0.12%). This systematic investigation of pharmacokinetic profiles of *arctigenin* (AG) *in vivo* and *in vitro* is worthy of further exploration.

## Introduction

**FIGURE 1:**
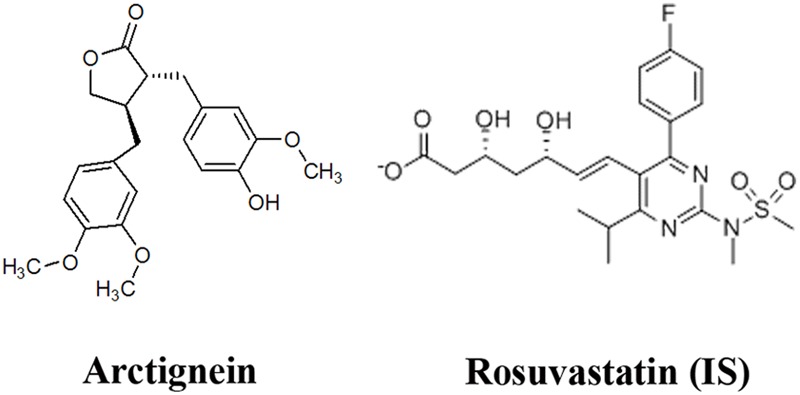
Chemical structures of arctigenin (AG) and rosuvastatin (IS).

Chinese traditional medicines (CTM), such as the fruit of *Forsythia suspensa Vahl, Ginkgo Biloba Extract* and *Forsythiae Fructus*, have been widely applied in the treatment of clinical diseases (Smith and Luo, 2004; Kang et al., 2008; Qu et al., 2008). The root of *Arctium lappa* L. as a popular edible vegetable in China and Japan, is used to be a general health tonic. In recent years, numerous studies have demonstrated that *A. lappa* (burdock) and its active components have multiple bioactivities *in vivo* and *in vitro* (Holetz et al., 2002; Liu et al., 2012; Hwangbo et al., 2014; Wu et al., 2014). Arctigenin (**Figure 1**) has been known to exert (1) Anti-oxidant activities: AG could decrease H_2_O_2_-induced ROS production and increase the antioxidant ability of the skeletal muscles (Wu et al., 2014); (2) anti-inflammatory activities: AG could inhibit pro-inflammatory factors, such as nuclear factor kappa B (NF-κB), inducible nitric oxide synthase, and oxidative stress (Liu et al., 2012; Hwangbo et al., 2014); (3) immunomodulatory activities: via inhibiting nitric oxide, interlukin-6 (IL-6) and tumor necrosis factor-α (TNFα) production in macrophages (Cho et al., 2004; Zhao et al., 2009); (4) anti-cancer activities: AG could block the unfolded protein response (UPR) and preferentially inhibit tumor cell viability under glucose deprived conditions (Kim et al., 2010; Sun et al., 2011; Lou et al., 2017; Maimaitili et al., 2017; Maxwell et al., 2017) and against leukemia cells (Matsumoto et al., 2006); and (5) neuroprotection activities: AG exerts a neuroprotective effect on both glutamate-induced neurotoxicity in primary neurons and scopolamine-induced learning and memory deficits in mice with Alzheimer’s disease (AD) (Jang et al., 2002; Lee et al., 2011; Song et al., 2016). *In vitro*, AG ameliorates memory impairment by suppressing microglia activation and decreasing IL-1β and TNF-α expression (Lee et al., 2011; Zhu et al., 2013).

Although numerous studies have discovered multiple promising pharmacological and extensively therapeutic activities of AG, systematic evaluation of its pharmacokinetic characteristics were still limited (Xin et al., 2015), although there are some ADME studies of this compound *in vivo* and *in vitro*. It is reported that AG can be metabolized to be arctigenic acid and arctigenin-4′-*O*-glucuronide in rats’ plasma by oral administration (Gao et al., 2014a). And there were different tissue distribution in SD rats (He et al., 2013). Different administration routes have great influence on drug metabolism. Here we evaluate effect of pharmaceutical dosage form, microemulsion, by different administration routes on AG metabolism.

In the current report, the plasma pharmacokinetic parameters in rats and beagle dogs were estimated *in vivo* after AG oral, i.h, s.l, and i.v administration. Subsequently, tissue distributions and urine, feces and bile excretions of AG were investigated. *In vitro*, to investigate whether the metabolic route is the predominant pathway for AG elimination, AG was incubated with each of human, monkey, beagle dog, and rat liver microsomes. The concentrations of AG were detected by UPLC-MS/MS at different time points (from 0 to 90 min).

## Materials and Methods

### Chemicals and Reagents

Highly pure (>99%) arctigenin was obtained from the State Key Laboratory of Generic Manufacture Technology of Chinese Traditional Medicine (*Lunan* Pharmaceutical Group Co. Ltd, Linyi, Shan Dong province, China). Rosuvastatin (internal standard) was obtained from Sigma (Sigma–Aldrich, St Louis, MO, United States). The structures of AG and rosuvastatin are shown in **Figure 1**. Methanol, formic acid and ammonium formate (chromatographic pure) were purchased from Fisher Scientific Co. (Fisher Scientific, Houston, TX, United States). A Milli-Q^®^ system was used to prepare Ultrapure HPLC-grade water (Millipore, Milford, MA, United States).

### Drug Administration and Sample Preparation

#### Ethics Statement

This study was carried out in compliance with the Chinese recommendations and legislation on laboratory animals use and care. The experiments protocols were approved by the Animal Ethics Committee of Lunan Pharmaceutical Group Co. Ltd.

#### Animals

For the plasma pharmacokinetics tissue distribution study, 48 Wistar rats (24 male and 24 female, 250–280 g, 10–12 weeks) and 48 Beagle dogs (10.0–12.45 kg, 24 male and 24 female) were supplied by Lunan Pharmaceutical Group Co. Ltd (Lin Yi, Shandong Province, China), acclimatized to a controlled temperature (23 ± 2°C) and maintained under a 12/12-h light/dark cycle. The animals were supplied with pellet chow and water *ad libitum*.

#### Pharmacokinetics Study

For the rats’ pharmacokinetics study, 18 rats were divided into an intragastric administration group (i.g), a hypodermic injection group (i.h), and an intravenous injection group (i.v), with six rats per group, to receive arctigenin (2.687 μmol/kg). For the dogs’ pharmacokinetics study, 48 beagle dogs were divided into sublingual administration groups (s.l, AG 2.687, 5.374, and 10.748 μmol/dog), hypodermic injection groups (i.h, AG 0.134, 0.403, and 1.209 μmol/kg) and intravenous injection groups (i.v, AG 5.374 μmol/dog and 0.403 μmol/kg, individually), with six beagle dogs per group. 0.1 ml of the dogs’ or rats’ jugular vein blood was obtained from 0 min to 12 h after AG administration. The blood samples were centrifuged at 4000 rpm for 10 min at 4°C to obtain the plasma (45 μl). Adding 5 μl internal standard (IS) and 145 μl methanol, centrifuging at 10000 rpm for 10 min at 4°C, and repeating this step once to obtain the supernatant, 100 μl samples were collected and AG concentrations were detected by HPLC-MS/MS.

#### Tissue Distribution Study

For the tissue distribution studies, 24 rats were hypodermically injected (i.h) with arctigenin (0.806 μmol/kg). After 0.25, 1, 3, and 6 h, all tissues, including the intestine, liver, heart, pancreas, kidney, plasma, stomach, muscle, ovary (female rats), lung, fat, spleen, brain, testis (male rats), uterus (female rats), and bone marrow, were separated and homogenized, and their AG concentrations were detected by UPLC-MS/MS.

#### *In Vitro* Binding and Microsome Stability Experiments

To determine AG binding in plasma, three different species of plasma (human, dog, and rat plasma) were used to dilute AG to varying drug concentrations (0.0672, 0.2687, and 1.075 μmol/L), using the validated 30 kDa ultrafiltration membrane method (Bekersky et al., 2002). Briefly, 350 μl plasma-drug samples were added to the upper ultrafiltration membrane tube (Merck Millipore, Billerica, MA, United States). 100 μl filtrates were collected and processed by UPLC-MS/MS after centrifuging for 30 min at 13,000 rpm.

For microsome stability experiments, incubations of AG in four different species of liver microsome (human, monkey, dog, and rat) were carried out as described previously (Gao et al., 2013). Briefly, 10 μl AG-methanol (100 μM) was pre-incubated with 50 μl liver microsome (20 mg/ml) in 840 μl 0.1 M PBS (PH 7.4) containing 50 μl MgCl_2_ (100 mM) for 5 min at 37°C, and the reaction was initiated by adding 50 μl NADPH regeneration system (XenoTech, LLC, Lenexa, KS, United States). 100 μl samples were collected from the reaction system at 0, 5, 10, 15, 20, 30, 60, and 90 min, respectively, and terminated by the addition of 100 μl of ice-cold methanol containing 80 μg/ml rosuvastatin (IS), followed by content analyses of AG by UPLC-MS/MS as described previously (Gao et al., 2014b). All the experiments were conducted in triplicate.

#### Urine, Feces, and Bile Excretion

For the urine and feces excretion assay, 12 rats were raised in metabolic cages and hypodermically injected with AG (0.806 μmol/kg). The urinary and fecal samples were collected at fixed time intervals from 0 to 2, 2 to 4, 4 to 6, 6 to 8, 8 to 12,12 to 24, 24 to 30, 30 to 48, 48 to 72 h. The total volume of urine and weight of feces were record and then stored at -80°C until analysis. For the bile excretion assay, six rats were anesthetized by i.p. injection of urethane (1.2 g/kg) and operated on by the bile duct-cannulation method. Then, the animals were administered AG (0.806 μmol/kg) via tail hypodermic injection. The bile samples were collected at the fixed time intervals from 0 to 2, 2 to 4, 4 to 6, 6 to 8, and 8 to 12 h, and all samples were stored at -80°C until analysis.

### Instrumentation for LC and MS

An ultra-performance liquid chromatography-electrospray ionization-mass spectrometer (UPLC-ESI-MS), containing a Waters ACQUITY UPLC system equipped with a photo diode array (PDA) (Waters, Milfora MA, United States) and Thermo ESI-mass spectrometer (Thermo Fisher Scientific, Inc., Waltham, MA, United States), was used. The sample solutions were separated using a Symmetry C_18_ column (4.6 mm × 150 mm, 5 μm) at 40°C. The following gradient system was used: mobile phase A (methanol containing 0.2% formic acid) and mobile phase B (10% methanol-water) were delivered at 0.6 ml/min; A:B 50:50 (v/v). The injection volume was 3 μl. Mass analysis by ESI was performed in positive mode. The spray voltage was 3500 V, the sheath gas (arbitrary unit, Arb) was 40, the aux gas (Arb) was 13, the sweep gas (Arb) was 1, the ion transfer tube temperature was 325°C, and the vaporizer temperature was 50°C. MS data were recorded in SRM mode, and arctigenin (m/z 373.2[M+H]^+^-m/z 137.3) and the IS rosuvastatin (m/z 482[M+H]^+^-m/z 258.2) were detected.

### Method Validation

#### Preparation of Standard Solutions

The AG solution was dissolved in methanol with a concentration of 100 μM. The desired concentrations from 0.2 to 500 ng/ml for AG were prepared by diluting the stock solution in methanol. Plasma or tissue calibrators were prepared individually by mixing 45 μl of the working standard solution with 5 μl IS and 145 μl methanol. Samples were then prepared as mentioned above.

#### Specificity and Matrix Effect

For the specificity assay, blank plasma and tissue homogenate samples were analyzed by UPLC-MS/MS and spiked with an AG standard, and rat plasma and tissue homogenate samples were analyzed after AG consumption. The processing procedure of all samples is mentioned above. The matrix effect was evaluated by comparing the corresponding peak area of the protein removed samples of blank plasma from six rats administrated AG with those of neat standard solutions at the same concentrations; this peak area ratio is defined as the matrix effect.

#### Linearity, Accuracy, and Precision

The calibration curves were established from the peak area of each standard solution against the nominal concentrations using eight level non-zero standards and a linearly weighed (1/x) least squares regression model. The calibration curve required a correlation coefficient (*R*^2^) of 0.99 or better.

Method accuracy was estimated by calculating the percent deviation observed in the analysis of Quality Control (QC) samples and expressed as relative error. Intraday precision was estimated by analyzing QC samples at three concentrations within 24 h (*n* = 5). Inter-day precision was estimated by repeated analysis of QC samples over three consecutive days (*n* = 15). The variability in determination was expressed as the relative standard deviation (RSD, %) and the accuracy was expressed as the relative error (RE, %). The LLOQ was defined as the lowest concentration that could be determined with both RE and RSD within 20% (Lee et al., 2011).

#### Recovery

The recoveries of AG from all samples were identified by comparing peak area ratios of regularly pretreated QC samples with those of the direct injection of pure standard solutions at three QC concentration levels. The recoveries of AG in all samples were examined at least three times.

#### Stability

The stabilities were determined by evaluating small variations under three different conditions. All stability studies were assayed at 50, 200, and 800 ng/mL. Short-term stability of AG for 24 h in different QC samples at room temperature was assayed. Long-term stability was studied by assaying samples storing for 2 weeks at -20°C. Freeze–thaw stability was detected after three consecutive freeze-thaw cycles (-20°C to room temperature). The results were showed as the percentage of the initial content of AG in the freshly treated samples. Samples were considered stable if assay values were within the acceptable limits of accuracy (±15% RE) and precision (±15% RSD).

### Data Analysis

Data were calculated and analyzed with Excel 2010 (Microsoft, Redmond, WA, United States) and SPSS V19.0 (SPSS, Inc., Chicago, IL, United States). Pharmacokinetic parameters, including *C*_max_, *T*_max_, and AUC0–∞, and the compartment model were analyzed by the Drug and Statistics (DAS) 3.0 software (Chinese Mathematical Pharmacology Society, Beijing, China). All values were presented as mean ± SD, using an unpaired *t*-test by SPSS Statistics V19.0.

## Results

### Selectivity and Matrix Effect

**FIGURE 2:**
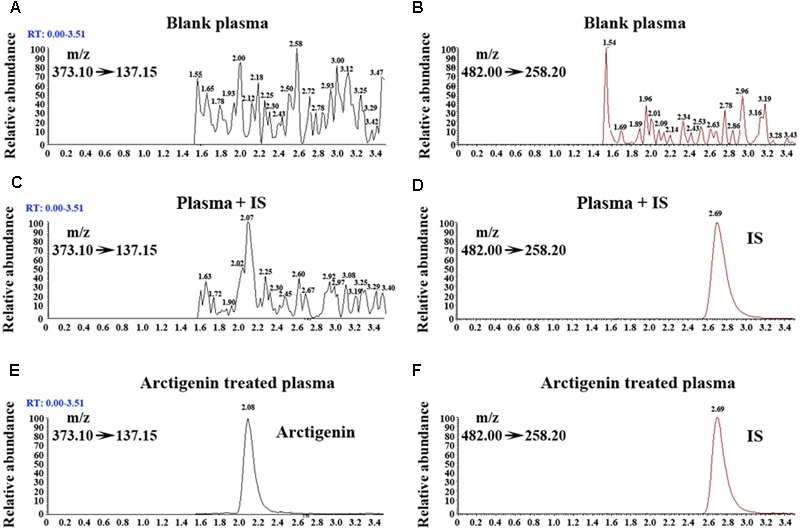
Representative extracted ion chromatograms of blank rats’ plasma sample spiked with AG **(A)** and IS **(B)** (MRM model). Rats’ plasma sample (with added IS) spiked with AG **(C)** and IS **(D)** (MRM model). Rats’ plasma sample (with added AG and IS) spiked with AG **(E)** and IS **(F)** (MRM model).

To evaluate the influence of endogenous interference, the peak area of blank plasma, blank plasma with added IS and blank plasma with added AG were compared with those of all samples spiked with AG and IS. The typical chromatograms of samples spiked with AG and IS are shown in **Figure 2**. In the presence of UPLC-MS/MS conditions described above, the retention time of AG was approximately 3.4 min. And there is no interference peak during the retention times in blank plasma, tissues, or even samples after AG consumption. The matrix effect values for AG at different levels were 111–126%, with RSD < 5.4%, suggesting that no significant matrix effects were found in plasma and tissue samples using this experimental conditions.

### Method Validation

#### Linearity and the Lower Limits of Quantitation

A linear relationship was found between the peak area and AG concentrations within the concentration ranges of 0.2–500 ng/mL. The coefficient of determination (*R*^2^) was found to be >0.99. The lower limit of quantitation (LLOQ) was 0.2 ng/ml for AG in plasma (data not shown).

#### Precision and Accuracy

The precision and accuracy were assessed by evaluating QC samples (*n* = 6) at three concentration levels, and the results are provided in **Table 1**. The accuracy of values ranged from -3.81 to 6.18%, and the intra- and inter-day precisions were 2.64 to 3.92% and 6.90 to 11.30%, respectively (data not shown). These results showed that this UPLC-MS/MS method was accurate, reliable, and reproducible.

**Table 1:** Pharmacokinetic parameters of arctigenin (2.687 μmol/kg) after intravenous (i.v) and hypodermic injection (i.h) AG administrations in Wistar rats (*n* = 6 per treatment group, results were presented as Mean ± SD).

Parameters	Units	i.h (dose)	i.v (dose)
		2.687 μmol/kg	2.687 μmol/kg
*C*_max_	μmol/L	1.26 ± 0.3	/
*T*_max_	Min	15 ± 0	/
AUC_0-t_	Min μmol/L	77.39 ± 22.9	66.26 ± 18.7
AUC_0-∞_	Min μmol/L	77.87 ± 24.67	67.87 ± 18.9
MRT_0-∞_	Min	54.48 ± 14.04	39.6 ± 7.38
MRT_0-t_	Min	49.86 ± 15.36	24 ± 6.26
Vd	L/kg	5.8 ± 2.13	12.4 ± 4.52
CL	L/min/kg	0.035 ± 0.011	0.04 ± 0.014
*t*_1/2_	Min	116.4 ± 21.72	217.8 ± 74.04
*F*		116%	

#### Recovery and Stability

The recoveries of AG at three different QC concentrations (*n* = 6) in plasma were 112–114%, and the recovery of IS was 95.6% (data not shown). It was found that the samples were stable after being at room temperature for 24 h (RE were from -6.27 to -0.25%), or stored at -20°C for 2 weeks (RE were from -8.23 to -4.50%), or even subjected to freeze–thaw cycles (RE were from 11.7 to 12%). In addition, the treated samples were stable in an auto sampler at 4°C for 60 h, and the RE ranged from 0.2 to 8.33%, which indicated that large scale samples could be detected in each analytical run. Therefore, a reliable, reproducible, and robust method has been developed and identified.

### Rats’ Plasma Pharmacokinetics Study

**FIGURE 3:**
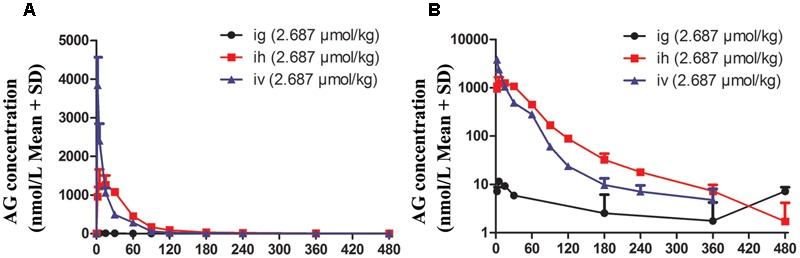
The plasma concentration vs. time profiles of arctigenin in rats after intragastric administration (i.g), hypodermic injection (i.h), and intravenous injection (i.v) arctigenin (2.687 μmol/kg). The results are shown using normal **(A)** or semilogarithmic coordinates **(B)**. (*n* = 6 per treatment group; results are presented as the mean ± SD).

To determine its pharmacokinetic characteristics, the plasma concentration-time profiles of AG were detected following i.h and i.v administrations of 2.687 μmol/kg of AG in rats (**Figure 3**), and the PK parameters are shown in **Table 1**. The results show that when rats under went hypodermic injection (i.h), the *C*_max_ and *T*_max_ were1.26 ± 0.3 μmol/L and 15 ± 0.0 min, respectively. The *T*_1/2_ in both the i.h and i.v groups were longer (116.4 ± 21.72 and 217.8 ± 74.04 min, respectively), which suggests a rather slow elimination process for AG after administrating the same dose of AG by i.h or i.v. Additionally, after i.g administration of 2.687 μmol/kg AG, the AG concentrations in rats’ plasma were detected at a series of times (0, 2, 5, 15, 30, 60, 90, 120, 180, 240, 360, and 480 min). However, unlike the results detected after i.h and/or i.v, only trace amounts of AG could be detected after i.g, and the concentrations were lower than LLOQ at most time points (**Figure 3**). All results suggest that oral administration of AG could influence the PK parameters and is not a suitable administration method. However, the *F*-value of AG was calculated to be 116%, indicating its higher i.h bioavailability.

### Beagle Dogs’ Plasma Pharmacokinetics Study

**FIGURE 4:**
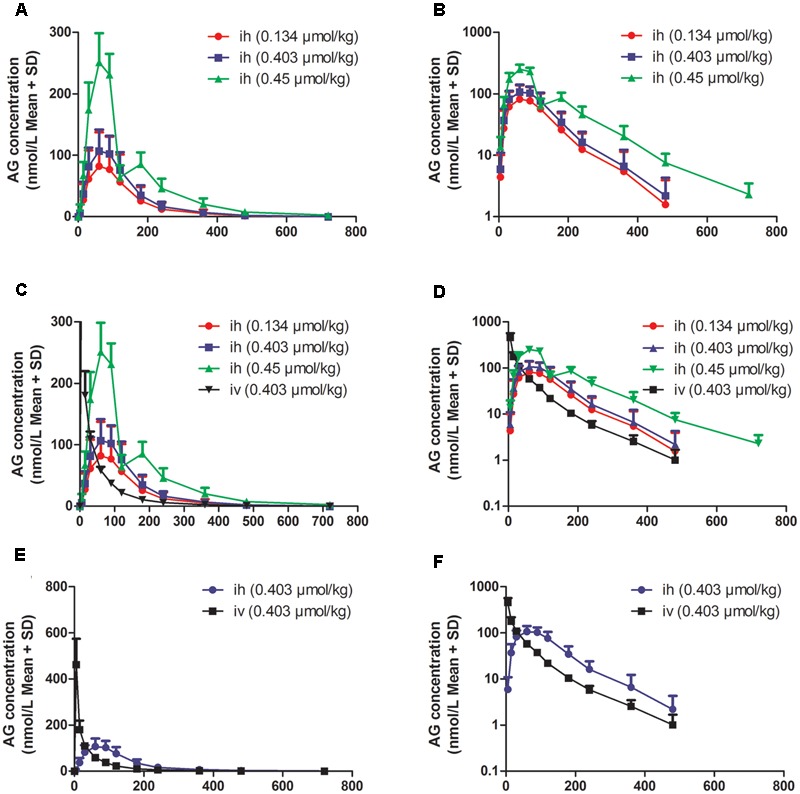
The plasma concentration vs. time profiles of arctigenin in beagle dogs after hypodermic injection (i.h) or intravenous injection (i.v) by arctigenin in different doses. **(A,B)** The different AG concentrations of beagle dogs’ plasma after i.h AG in 0.134, 0.403, or 0.45 μmol/kg. **(C,D)** The comparison between i.h and i.v of AG concentration. **(E,F)** The comparison between i.h and i.v of AG concentration at the dose of 0.403 μmol/kg. The results are shown using normal (left) or semilogarithmic coordinates (right). (*n* = 6 per treatment group; results are presented as the mean ± SD).

To further investigate the pharmacokinetic characteristics of AG, beagle dogs were administered AG by i.h and i.v. To eliminate the influence of bioavailability after oral administration, dogs were also administered AG by sublingual administration (s.l), and the plasma concentrations of AG were detected at a series of times (from 0 min to 12 h). **Figure 4** and **Table 2** show the plasma concentration-time profiles and PK parameters of AG after i.h administrations of 0.134, 0.403, and 1.209 μmol/kg AG in beagle dogs. After i.h administration of AG at 0.134, 0.403, and 1.209 μmol/kg, the *C*_max_ were 0.032 ± 0.005, 0.113 ± 0.03, and 0.252 ± 0.04 μmol/L, respectively. The *T*_max_ were 60, 70.2 ± 15.48 and 75 ± 16.44 min, respectively, with no significant differences between the three dose groups (**Figures 4A,B** and **Table 2**). In addition, the *T*_1/2_ in all three groups was longer than 1 h (94.8 ± 31.74, 74.4 ± 12.18, and 117 ± 25.44 min, respectively), which indicated a rather slow elimination process for AG after administrating the different doses of AG by i.h. Compared with i.h administration, i.v AG showed a long *T*_1/2_ (96 ± 24.84 min). Finally, under the same dose of AG (0.403 μmol/kg), the AUC_0-t_ and AUC_0-∞_ showed no significant difference in both the i.h and i.v groups, which suggests that AG has a rather high bioavailability after i.h administration (**Figures 4C–F** and **Table 2**).

**Table 2:** Pharmacokinetic parameters of arctigenin after intravenous (i.v) and hypodermic injection (i.h) AG administrations in beagle dogs (*n* = 6 per treatment group, results were presented as Mean ± SD).

Parameters	Units	i.h (dose)			i.v (dose)
		0.134 μmol/kg	0.403 μmol/kg	1.209 μmol/kg	0.403 μmol/kg
*C*_5__min_	μmol/L	/	/	/	0.462 ± 0.11
*C*_max_	μmol/L	0.032 ± 0.005	0.113 ± 0.03	0.252 ± 0.04	/
*T*_max_	Min	60	70.2 ± 15.48	75 ± 16.44	/
AUC_0-t_	Min μmol/L	4.32 ± 0.54	16.61 ± 5.27	35.31 ± 4.13	15.45 ± 2.02
AUC_0-∞_	Min μmol/L	4.76 ± 0.75	16.93 ± 5.42	35.63 ± 4.21	15.64 ± 2.08
MRT_0-∞_	Min	123.6 ± 14.52	120 ± 20.52	144.6 ± 23.88	53.04 ± 3.55
MRT_0-t_	Min	158.4 ± 54.66	127.2 ± 26.46	153 ± 25.80	59.64 ± 5.02
Vd	L/kg	3.85 ± 0.88	2.74 ± 0.72	5.87 ± 1.88	3.54 ± 0.56
CL	L/min/kg	0.029 ± 0.05	0.026 ± 0.007	0.034 ± 0.004	0.026 ± 0.004
*t*_1/2_	Min	94.8 ± 31.74	74.4 ± 12.18	117 ± 25.44	96 ± 24.84
*F*			108%		

**FIGURE 5:**
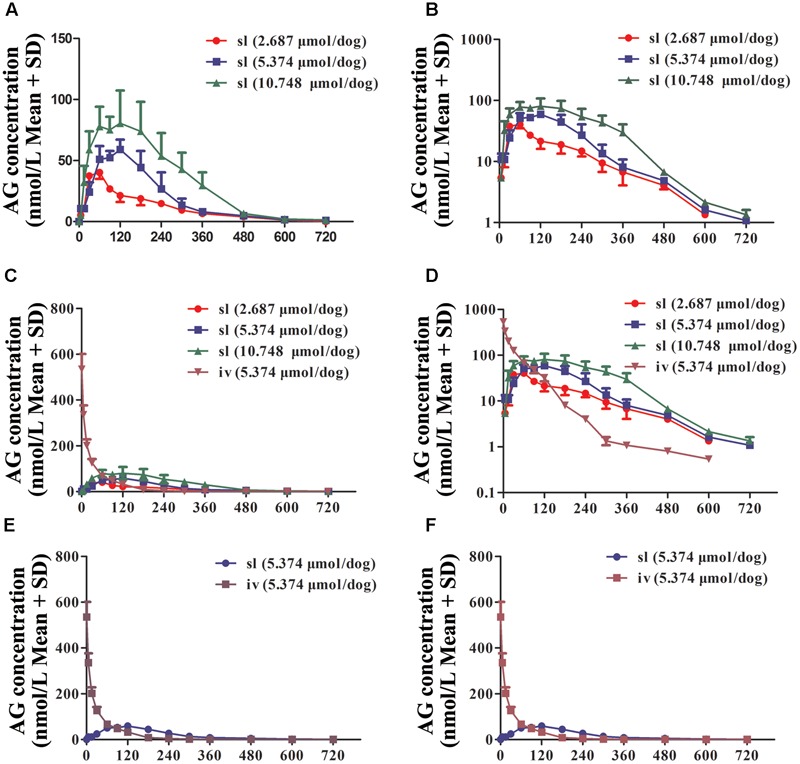
The plasma concentration vs. time profiles of arctigenin in beagle dogs after sublingual administration (s.l) or intravenous injection (i.v) by arctigenin in different doses. **(A,B)** The different AG concentrations of beagle dogs’ plasma after i.h AG in 2.687, 5.374, or 10.748 μmol/dog. **(C,D)** The comparison between s.l and i.v of AG concentration. **(E,F)** The comparison between s.l and i.v of AG concentration at the dose of 5.374 μmol/dog. The results showed as normal (left) or semilogarithmic coordinates (right). (*n* = 6 per treatment group; results are presented as the mean ± SD).

To eliminate the low AG bioavailability induced by oral administration, beagle dogs were administered AG by sublingual administration (s.l) at the doses of 2.687, 5.374, and 10.748 μmol/dog. To determine the PK characters of s.l, dogs accepted i.v of AG at the dose of 5.374 μmol/dog. In all groups, the plasma AG concentrations at different time points were detected and calculated (**Figure 5** and **Table 3**). As shown in **Figure 5**, s.l AG still exhibited a slow elimination process because of the long *T*_1/2_ in all three dose groups (**Figures 5A,B**). The *F*-value of AG was calculated to be 72.5%, indicating its higher s.l bioavailability.

**Table 3:** Pharmacokinetic parameters of arctigenin after intravenous (i.v) and sublingual (s.l) AG administrations in beagle dogs (*n* = 6 per treatment group, results were presented as Mean ± SD).

Parameters	Units	s.l (dose)			i.v (dose)
		2.687 μmol/dog	5.374 μmol/dog	10.748 μmol/dog	5.374 μmol/dog
*C*_5 min_	μmol/L	/	/	/	0.53 ± 0.085
*C*_max_	μmol/L	0.04 ± 0.007	0.07 ± 0.24	0.1 ± 0.018	/
*T*_max_	Min	60 ± 16.44	112 ± 53.64	130.2 ± 70.20	/
AUC_0-t_	Min μmol/L	6.48 ± 1.13	11.93 ± 2.27	23.22 ± 3.10	16.44 ± 1.51
AUC_0-∞_	Min μmol/L	6.56 ± 1.16	12.01 ± 2.26	23.22 ± 3.06	16.61 ± 1.50
MRT_0-∞_	Min	162 ± 31.5	167.4 ± 21.36	195.6 ± 36.42	72 ± 11.94
MRT_0-t_	Min	168 ± 34.8	171 ± 23.16	198.6 ± 35.34	75 ± 12.42
Vd	L/kg	36.3 ± 8.22	46.3 ± 14.6	58.5 ± 29.3	36.1 ± 8.45
CL	L/min/kg	0.42 ± 0.09	0.46 ± 0.01	0.47 ± 0.06	0.33 ± 0.028
*t*_1/2_	Min	61.8 ± 19.26	70.8 ± 24.36	84 ± 34.98	96 ± 24.84
*F*			72.5%		

### The Tissue Distributions of AG in Rats

**FIGURE 6:**
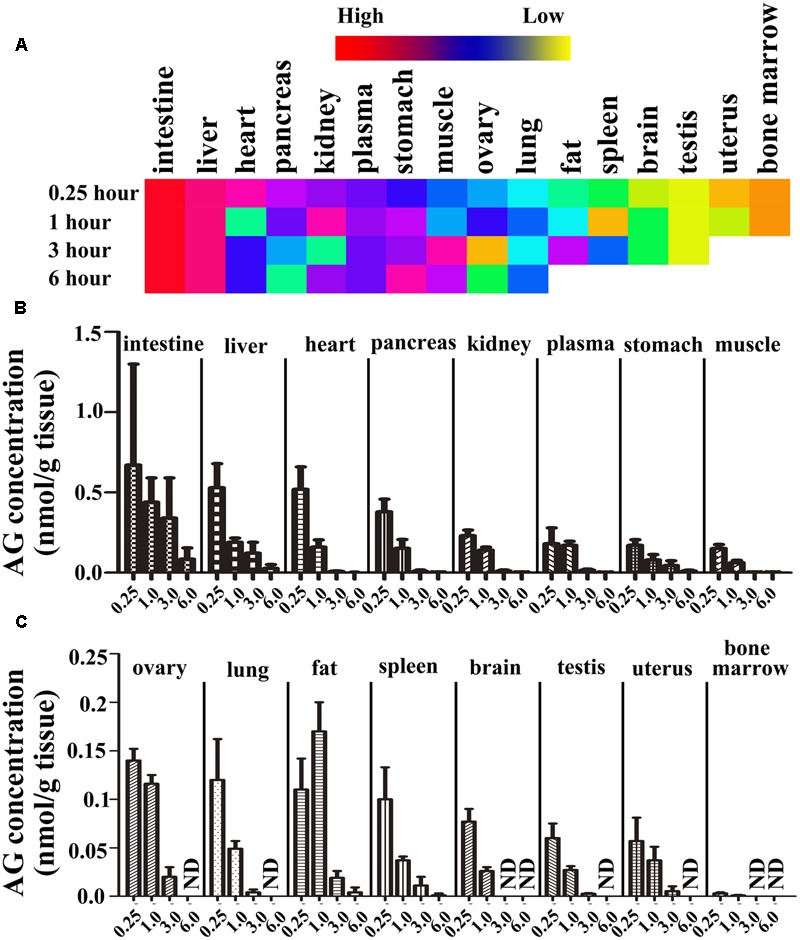
The tissue distribution of arctigenin in rats. **(A)** The heat map representing the concentration of arctigenin in different tissues after rats were hypodermically injected with 8.061 μmol/L. **(B,C)** The histogram representing the tissue distributions of arctigenin in rats. (ND means not detected. *N* = 6 per treatment group; results are presented as the mean ± SD).

To determine the tissue distributions of AG, rats were hypodermically injected with 8.061 μmol/L AG, and after 0.25, 1, 3, and 6 h, all tissues, including intestine, liver, heart, pancreas, kidney, plasma, stomach, muscle, ovary (female rats), lung, fat, spleen, brain, testis (male rats), uterus (female rats), and bone marrow, were separated and homogenized, and their AG concentrations were detected by UPLC-MS/MS. As shown in **Figure 6** and **Table 4**, the data suggest that the AG is widely distributed throughout all tissues and even the brain, testis (or uterus) and bone marrow (**Figure 6A**). At 0.5 h after AG consumption, the intestinal, liver and heart concentrations of AG peaked; the pancreas, kidney, plasma, and stomach contained moderate amounts of AG; and low concentrations of AG was detected in tissue extracts from the muscle, ovary, lung, fat, spleen, brain, testis, uterus, and bone marrow. At 6 h post-dosing, concentrations of AG was still comparatively high in tissue extracts from the intestine and liver, whereas extracts from the fat, spleen, brain, testis (male rats), uterus (female rats), and bone marrow had relatively low concentrations of AG. Notably, at 0.5 h after administration, AG in the brain was detected at a relatively low concentration, and the concentration increased from 1 to 3 h, suggesting that AG could cross the blood–brain barrier. However, at 6 h after administration, AG was not detected in almost organs and tissues, e.g., the fat, spleen, brain, testis, uterus, and bone marrow, but it could be still detected in intestine and liver.

**Table 4:** The tissue distribution of arctigenin (0.806 μmol/kg) on rats.

Tissues/Plasma	AG concentration (nmol/g)				AUC_0-6 h_
	0.25 h	1 h	3 h	6 h	(nmol⋅h/g)
Plasma^∗^	0.18 ± 0.099	0.17 ± 0.025	0.016 ± 0.0059	0.002 ± 0.001	0.39
Liver	0.53 ± 0.15	0.19 ± 0.025	0.122 ± 0.068	0.027 ± 0.023	0.86
Heart	0.52 ± 0.14	0.16 ± 0.045	0.008 ± 0.003	0.001 ± 0.002	0.49
Lung	0.12 ± 0.042	0.049 ± 0.008	0.004 ± 0.003	/	0.14
Kidney	0.23 ± 0.035	0.14 ± 0.02	0.011 ± 0.006	0.002 ± 0.003	0.33
Spleen	0.1 ± 0.033	0.037 ± 0.004	0.011 ± 0.009	0.001 ± 0.002	0.13
Testis	0.06 ± 0.015	0.027 ± 0.004	0.0026 ± 0.0006	/	0.074
Brain	0.077 ± 0.013	0.026 ± 0.004	/	/	0.075
Intestines	0.67 ± 0.63	0.44 ± 0.15	0.34 ± 0.25	0.084 ± 0.07	1.91
Stomach	0.17 ± 0.036	0.08 ± 0.033	0.044 ± 0.03	0.011 ± 0.004	0.32
Muscle	0.15 ± 0.025	0.062 ± 0.015	0.0037 ± 0.001	0.0019 ± 0.002	0.17
Fat	0.11 ± 0.032	0.17 ± 0.03	0.019 ± 0.007	0.0041 ± 0.005	0.34
Uterus	0.057 ± 0.024	0.037 ± 0.014	0.0052 ± 0.0051	/	0.092
Pancreas	0.38 ± 0.078	0.152 ± 0.055	0.01 ± 0.007	0.0024 ± 0.002	0.43
Bone Marrow^∗^	0.003 ± 0.001	0.0009 ± 0.0005	/	/	0.0027
Ovary	0.14 ± 0.012	0.116 ± 0.009	0.02 ± 0.01	/	0.28

*^∗^In plasma and bone marrow, the concentration Units is nmol/mL.*

*Twenty-four rats hypodermic injected (i.h) arctigenin (0.806 μmol/kg) and all tissues were collected at 0.25, 1, 3, and 6 h after injection and the arctigenin concentrations were detected by LC-MS/MS. (*n* = 6 per treatment group, results were presented as Mean ± SD).*

### Plasma Protein Binding and Microsome Stability of AG

To determine the plasma protein binding and microsome stability of AG, plasma (from human, beagle dog, and rat) and liver microsomes (from human, monkey, rat, and dog) were used. In the plasma protein binding assay, AG was diluted by human, beagle dog, or rat plasma in different concentrations. As the results shown (**Table 5**), when AG was present at different concentrations (0.0672, 0.269, and 1.075 μM), the plasma protein binding rates of AG in human or rat plasma were almost 100%. Furthermore, the binding rates of AG in beagle dogs were 100, 99.9, and 99.8% in different concentrations. The results indicate that AG exhibits a strong binding capacity with plasma, including human, beagle dog, and rat. The protein binding capacity of AG showed no significant difference between human, dog, and rat plasma.

**Table 5:** The plasma protein binding rate of AG.

AG concentration (μmol/mL)	Plasma protein binding rate (%)		
	Rat	Dog	Human
0.0672	100 ± 0	100 ± 0	100 ± 0
0.2687	100 ± 0	99.9 ± 0.0198	100 ± 0
1.075	100 ± 0	99.8 ± 0.0336	100 ± 0
Mean	100 ± 0	99.9 ± 0.0178	100 ± 0

*AG were prepared to three different concentration (0.0672, 0.2687, and 1.075 μmol/L) by human, rat, and dog fresh plasma, individually. All samples (350 μl) added into ultrafiltration tube, 13000 rpm, 30 min, filtrate (100 μl) were detected by LC-MS/MS. (Triplicate assays were performed and results were presented as Mean ± SD).*

**FIGURE 7:**
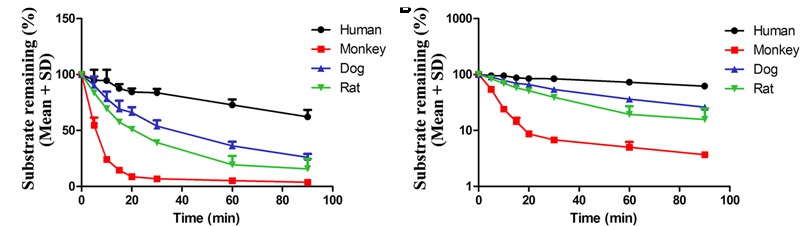
The stability of AG in four species’ liver microsomes. 10 μl AG (10 μM) incubated with different liver microsome and concentration of AG were detected at 0, 5, 10, 15, 20, 30, 60, and 90 min by UPLC-MS/MS, respectively. The results are shown using normal **(A)** or semilogarithmic coordinates **(B)** (*n* = 6 per treatment group; results are presented as the mean ± SD).

To study the stability of AG *in vitro*, four species of liver microsomes, including human, monkey, beagle dog, and rat, were used. The remaining substrates in reaction systems at different time points were shown in **Table 6**. The results showed that the metabolic stability of AG exhibited a species difference in different liver microsomes (**Figure 7**).

**Table 6:** The metabolic distinguish of AG in different species.

Time (min)	Remaining substrate ratio (%)			
	Human	Monkey	Dog	Rat
0	100 ± 0	100 ± 0	100 ± 0	100 ± 0
5	94.8 ± 9.33	54.6 ± 6.86	90.3 ± 7.88	83.5 ± 2.58
10	94.6 ± 9.61	24.1 ± 1.46	78.4 ± 6.32	69.4 ± 2.21
15	87.4 ± 3.93	14.4 ± 2.38	69.2 ± 7.46	57.6 ± 2.76
20	84.3 ± 3.10	8.67 ± 1.00	66.0 ± 4.69	51 ± 2.63
30	83.7 ± 3.34	6.78 ± 0.38	53.9 ± 5.06	39.1 ± 2.76
60	72.8 ± 4.83	5.00 ± 1.21	36.2 ± 3.68	19.3 ± 7.89
90	62 ± 6.36	3.69 ± 0.12	25.9 ± 3.21	15.7 ± 9
	
***k*_e_**	0.00508	0.124	0.0167	0.027
**t1/2 (min)**	136	5.59	41.5	25.7

*Ten microliter AG (100 μM) incubated with different microsome (human, monkey, dog, and rat) at 37°C for 3 min. Hundred microliter samples were collected at 0, 5, 10, 20, 30, 60, and 90 min and detected by LC-MS/MS. (Triplicate assays were performed and results were presented as Mean ± SD).*

### The Excretion of AG in Rats

**FIGURE 8:**
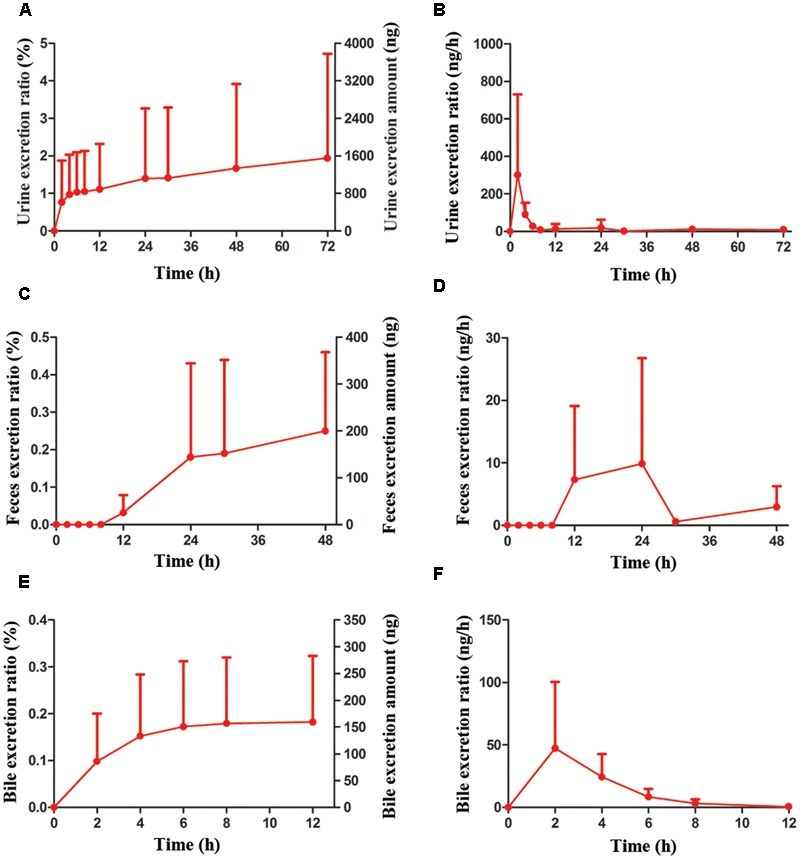
The accumulative excretions and excretion rate of AG (0.806 μmol/kg, i.h) on Wistar rats in different times. **(A,B)** The urine excretion ratio and amount of AG in rats. **(C,D)** The feces excretion ratio and amount of AG in rats. **(E,F)** The bile excretion ratio and amount of AG in rats. (*n* = 6 per treatment group; results are presented as the mean ± SD).

To detect the excretion characteristics of AG, Wistar rats were hypodermically injected with 0.806 μmol/kg of AG. Urine, feces and bile were collected at different time points and detected by UPLC-MS/MS. The data were calculated and shown in Supplementary Table 1. The urine excretion results indicate that 72 h after AG administration, the accumulated excretion amount of AG in rats’ urine was 1557 ± 2165 ng, and the excretion ratiowas1.94 ± 2.78% (**Figures 8A,B**). In the first 8 h and beyond 24 h after AG administration, AG cannot be detected in rat feces (Supplementary Table 1). The accumulated excretion amount of AG in rats’ urine 48 h after administration was 204 ± 166 ng, and the excretion ratio was 0.25 ± 0.21% (**Figures 8C,D**). For bile excretion, the bile was collected from 0 to 12 h and measured. The data showed that the accumulated excretion amount of AG in rats’ bile was 169 ± 145 ng, and the excretion ratio was 0.182 ± 0.141% (**Figures 8E,F**).

## Discussion

In this research, rats and beagle dogs were used to investigate the PK profiles of AG with different drug-delivery methods, including intravenous (i.v), hypodermic injection (i.h), and sublingual (s.l) administration. The data shows that AG exhibited a strong absorption capacity in both rats and beagle dogs (absorption rate < 1 h), a high absorption degree (absolute bioavailability > 100%), and a strong elimination ability (*t*_1/2_ < 2 h). It is reported that AG underwent rapid hydrolysis in plasma in SD rats. And in intestine and liver, AG could be extensively metabolized to one mono-glucuronide and undergo a first-pass metabolism during the absorption process. Therefore, elimination ability is strong (Gao et al., 2014a,b; Xin et al., 2015).

Furthermore, we used Wistar rats to investigate the tissue distributions of AG at different time points after i.h and used human, beagle dog, and rat plasma to assay the plasma protein binding capacity of AG. The results of the AG tissue distributions showed that the concentrations of AG at 0.5 h were intestine > liver > heart > pancreas > kidney > plasma > stomach > muscle > ovary (female rats) > lung > fat > spleen > brain > testis (male rats) > uterus (female rats) > bone marrow; at 1 h were intestine > liver > fat > plasma > heart > kidney > pancreas > ovary (female rats) > stomach > muscle > lung > uterus (female rats) > spleen > testis (male rats) > brain > bone marrow; and at 3 h were intestine > liver > stomach > ovary (female rats) > fat > plasma > kidney > heart > uterus (female rats) > lung > pancreas > muscle > spleen > testis (male rats), with AG not detected in the brain and bone marrow. The AG contents at 6 h in different tissues were intestine > liver > stomach > fat > kidney > plasma > heart > pancreas > spleen > muscle, ovary (female rats) > uterus (female rats) > lung > testis (male rats); AG was not detected in the other tissues. All these results indicate that the distribution of AG in rat tissues is rapid (2.5 h to reach the peak) and wide (could detect it in almost all tissues and organs). Meanwhile, its elimination is also rapid because, except in the intestine, the concentrations of AG were reduced at 6 h to 1/10 of their values 0.25 h after drug-delivery, implying that there is no accumulation in tissues and organs after i.h of AG. Compared with other tissues, the AG concentration in the intestine was the highest, and relatively higher in the heart, liver, pancreas, and kidney, which are rich in blood supply. In fat, the distribution of AG reached its peak at 1 h and the elimination was slow, suggesting that this may relate to the lipid solubility of AG. In this study, concentration of AG in intestine and liver was high, which may be due to may be due to the highly perfused nature of both intestine and liver. He et al. (2013) found that concentration of AG in spleen was the highest in SD rats by oral administration, which might be due to different route of administration and different pharmaceutical dosage form. It was found that AG was stable in both gastric and intestinal fluids (Gao et al., 2014a), which was contributed to the high concentration in intestine and stomach. And our results showed that *t*_1/2_ of AG in Wistar rats by i.v. was different from Gao’s results, which might due to the pharmaceutical dosage form. Microemulsion formulations of AG extremely enhanced the half life of AG. Phase I trial of GBS-01, an orally administered drug rich in arctigenin, showed that *t*_1/2_ of AG was influenced by dose of the drug (Ikeda et al., 2016), from 3 to 7 h. And it was found that s.l administration was a better method than the other two. This administration route could be easy to carry out for patients in the clinic. It was found that the stability of AG was good at room temperature and -20°C. This result was in accordance with He’s report (He et al., 2013).

Subsequently, we investigated the urine, feces and bile excretions of AG in rats after i.h of AG. Within 72 h after drug-delivery, the urine accumulative excretion ratio was 1.93%, and the ratio reached a platform after 12 h, resulting from its high lipophilic and plasma protein binding capacity. Discharge from bile into intestines and intestinal autocrine are the predominant mechanisms when drugs excrete via feces. In this experiment, AG was not detected in the feces within 8 h after AG administration, and the excretory amount of AG in feces and bile were 0.248 and 0.182%, respectively. These data imply that the main elimination pathways of AG in rats are through metabolism.

To investigate whether the metabolic route is the predominant pathway for AG elimination, the AG were incubated with human, monkey, beagle dog, and rat liver microsomes, respectively. The concentrations of AG were detected by UPLC-MS/MS at different time points (from 0 to 90 min). The substrates remaining of AG in four species liver microsomes were human (62 ± 6.36%) > beagle dog (25.9 ± 3.24%) > rat (15.7 ± 9%) > monkey (3.69 ± 0.12%). It was reported that AG could be metabolized to one mono-glucuronide in human liver microsomes (Xin et al., 2015). And AG could be metabolized to arctigenic acid and arctigenin-4′-*O*-glucuronide by the first pass effect in intestine of rats (Gao et al., 2014a,b).

## Conclusion

Although AG has been reported to possess multiple biological activities and to be involved in anti-oxidative activities, inhibition angiogenesis and even anti-cancer activities, the pharmacokinetic profiles of AG have garnered less attention. This study providing a systematic investigation of the pharmacokinetic profiles of *arctigenin* (AG) *in vivo* and *in vitro* is worthy of further exploration.

## Author Contributions

G-MZ and ZL contributed to the conception and design of the study and approved the final version to be submitted. JL contributed to drafting the article and data analysis and interpretation. XL, Y-SR, and J-CY performed sample collection from experimental animals. Y-YL, J-SZ, X-LX, and X-ZW performed sample detection.

## Conflict of Interest Statement

The authors declare that the research was conducted in the absence of any commercial or financial relationships that could be construed as a potential conflict of interest.
