# Supplementary material for: Elucidation of Arctigenin Pharmacokinetics and Tissue Distribution after Intravenous, Oral, Hypodermic and Sublingual Administration in Rats and Beagle Dogs: Integration of In Vitro and In Vivo Findings
Source: Front Pharmacol. 2017 Jun 14;8:376. doi: 10.3389/fphar.2017.00376 (PMC5469903; doi:10.3389/fphar.2017.00376)
Supplement: Supplementary file 1 [file Table_1.DOC]

**Supplementary material**

**Supplementary Table 1. The accumulative excretions and excretion rate of AG (0.806 μmol/kg, i.h) on Wistar rats in different times.** Rats were hypodermic injected (i.h) by arctigenin (0.806 μmol/kg). The urine, feces and bile were collected in different times. All samples were detected by LC-MS/MS. (n=6 per Treatment Group, Results Were Presented as Mean ± SD).

| **Time** |  |  | **Urine** | | | |  |  |  | **Feces** | | | |  |  |  | **Blie** | | |  |
| --- | --- | --- | --- | --- | --- | --- | --- | --- | --- | --- | --- | --- | --- | --- | --- | --- | --- | --- | --- | --- |
| **(hour)** | **EA(ng)** | | | **AEA (ng)** | **ERo (%)** | **ER (ng/h)** | | **EA(ng)** | | | **AEA (ng)** | **ERo (%)** | **ER (ng/h)** | | **EA(ng)** | | | **AEA (ng)** | **ERo (%)** | **ER (ng/h)** |
| **0** | 0 ± 0 | | | 0 ± 0 | 0 ± 0 | 0 ± 0 | | 0 ± 0 | | | 0 ± 0 | 0 ± 0 | 0 ± 0 | | 0 ± 0 | | | 0 ± 0 | 0 ± 0 | 0 ± 0 |
| **2** | 600 ± 863 | | | 600 ± 863 | 0.765 ± 1.11 | 300 ± 431 | | 0 ± 0 | | | 0 ± 0 | 0 ± 0 | 0 ± 0 | | 94.7 ± 107 | | | 94.7 ± 107 | 0.098 ± 0.102 | 47.3 ± 53.3 |
| **4** | 181 ± 124 | | | 781 ± 816 | 0.967 ± 1.07 | 90.7 ± 61.9 | | 0 ± 0 | | | 0 ± 0 | 0 ± 0 | 0 ± 0 | | 48.8 ± 36.7 | | | 143 ± 138 | 0.152 ± 0.132 | 24.4 ± 18.4 |
| **6** | 56 ± 23.6 | | | 837 ± 814 | 1.03 ± 1.07 | 28 ± 11.8 | | 0 ± 0 | | | 0 ± 0 | 0 ± 0 | 0 ± 0 | | 16.9 ± 12.4 | | | 160 ± 145 | 0.172 ± 0.140 | 8.45 ± 6.20 |
| **8** | 14.9 ± 12.6 | | | 852 ± 824 | 1.05 ± 1.08 | 7.46 ± 6.32 | | 0 ± 0 | | | 0 ± 0 | 0 ± 0 | 0 ± 0 | | 6.47 ± 6.50 | | | 167 ± 146 | 0.179 ± 0.141 | 3.23 ± 3.25 |
| **12** | 51.5 ±104 | | | 903 ± 926 | 1.11 ± 1.21 | 12.9 ± 26 | | 29.2 ±47.2 | | | 29.2 ±47.2 | 0.032 ± 0.047 | 7.3 ± 11.8 | | 2.42 ± 3.96 | | | 169 ± 145 | 0.182 ± 0.141 | 0.606 ± 0.99 |
| **24** | 225 ± 527 | | | 1129 ± 1447 | 1.40 ± 1.87 | 18.8 ± 43.9 | | 118 ± 202 | | | 147 | 0.18 ± 0.25 | 9.86 ± 16.9 | | / | | | / | / | / |
| **30** | 7.56 ± 10.7 | | | 1136 ± 1452 | 1.41 ± 1.88 | 1.26 ± 1.78 | | 3.490 ± / | | | 151 | 0.19 ± 0.25 | 0.581 ± / | | / | | | / | / | / |
| **48** | 206 ± 333 | | | 1342 ± 1736 | 1.67 ± 2.24 | 11.4 ± 18.5 | | 53.10 ± 59.6 | | | 204 | 0.25 ± 0.21 | 2.95 ± 3.31 | | / | | | / | / | / |
| **72** | 215 ± 433 | | | 1557 ± 2165 | 1.94 ± 2.78 | 8.97 ± 18.1 | | / | | | / | / | / | | / | | | / | / | / |

EA, Excretion Amount. AEA, Accumulated excretion amount. ERo, Excretion Ratio. ER, Excretion Rate
